# Diverting ileostomy for treatment of ileoanal pouch dysfunction: a technical note

**DOI:** 10.1007/s00384-024-04756-y

**Published:** 2024-11-16

**Authors:** Serena Weng, Orsalia Mangana, Pietro Calabrese, Valerio Celentano

**Affiliations:** 1 Bowel Disease and Ileoanal Pouch Surgery Centre, Chelsea and Westminster Hospital, London, UK; 2 Division of Surgery and Cancer, Imperial College London, London, UK; 3 Department of General Surgery, Transplantation and Gastroenterology, Federico II University Hospital, Naples, Italy; 4 Université Paris Cité, Paris, France

**Keywords:** Ulcerative colitis, Ileoanal pouch, Dysfunction, Diverting ileostomy, Laparoscopic, Pouchoscopy

## Abstract

**Background:**

The ileal pouch-anal anastomosis (IPAA) is a restorative procedure performed after proctocolectomy to improve quality of life in patients with colorectal conditions like ulcerative colitis, familial adenomatous polyposis, and selected cases of Crohn’s disease and Lynch syndrome. However, severe pouch dysfunction can occur, often necessitating further surgical intervention.

**Objective:**

This technical note aims to describe the operative approach and perioperative management for diverting ileostomy as a treatment for dysfunctional ileoanal pouches.

**Methods:**

Indications for the procedure include complications such as pelvic sepsis, pouchitis, fistulas, and Crohn’s disease of the pouch. Preoperative planning involves a multidisciplinary team, stoma site marking, and imaging to assess bowel integrity. The surgical technique utilizes laparoscopic access with careful adhesiolysis to minimize bowel injury, with intraoperative pouchoscopy to identify anatomical landmarks. An ileostomy is created by selecting a tension-free small bowel segment and approximating it to a pre-marked stoma site. Attention is given to preserving bowel length to allow for potential future restorative procedures. Postoperative care focuses on stoma management and addressing ongoing pouch dysfunction symptoms.

**Conclusions:**

Diverting ileostomy offers symptom relief for patients with pouch dysfunction while avoiding more complex procedures like pouch excision. It is a valuable option in managing pouch failure.

**Supplementary Information:**

The online version contains supplementary material available at 10.1007/s00384-024-04756-y.

## Introduction

The ileal pouch-anal anastomosis (IPAA) is a restorative procedure performed after proctocolectomy, offering improved quality of life for patients with colorectal conditions such as ulcerative colitis and familial adenomatous polyposis and selected cases of Crohn’s disease and Lynch syndrome. Although many patients with IPAA have high long-term satisfaction, severe pouch dysfunction leading to the need for pouch excision or permanent ileostomy occurs in approximately 5–15% of cases. The unfortunate wording of pouch failure is often described in the literature and is defined as the need for a permanent ileostomy or pouch excision due to complications.

Several complications can lead to pouch failure, including anastomotic leakage, pelvic sepsis, fistulas, strictures, recurrent pouchitis and Crohn’s disease (CD) of the pouch. Among these, pelvic sepsis is the most significant risk factor for pouch failure, with an associated need for salvage surgery in approximately 50% of cases. Other causes include mechanical complications (44.6%), such as poor emptying, pouch ischemia or pouch prolapse, and less commonly neoplastic causes (0.3%) [1]. Various risk factors, such as obesity (BMI > 30), indeterminate colitis or Crohn’s disease and the use of immunosuppressive medications at the time of IPAA, have been associated with an increased risk of pelvic sepsis, further complicating outcomes [2].

The surgical management of pouch dysfunction varies significantly depending on the underlying cause. The main options include pouch excision, permanent diversion with the pouch left in situ, revisional and redo IPAA surgery.

Permanent diversion with the pouch left in situ is a less invasive alternative that avoids the more extensive procedure of pouch removal. While this approach spares the patient from a complex surgery, complications related to the retained pouch, such as mucous discharge, pouchitis or pelvic sepsis, may still occur. This strategy is often employed when the patient is not a candidate for pouch excision due to extensive adhesions or other surgical contraindications, or more frequently, as a bridge towards a more radical excisional or revisional IPAA surgery. The aim of this technical note is to describe the operative approach for diversion of the dysfunctional ileoanal pouch and pre and postoperative management.

## Methods

### Preoperative preparation

A multidisciplinary team and dedicated pathway are necessary for the management of patients with pouch dysfunction [3].

Patient symptoms, quality of life and expectations play a pivotal role in making such a complex decision of treating with surgery a poorly behaving ileoanal pouch. In preparation for surgery is particularly important to review the previous operating notes and endoscopic, radiological and pathological results. Several outpatient clinical reviews and discussions are often needed prior to proceeding to surgery. Peer-support from patients’ groups and psychological counselling need to be integrated in the care pathway [3].

Preoperative preparation for diverting ileostomy in patients with pouch dysfunction involves several critical steps aimed at minimizing complications and ensuring optimal outcomes. These steps include:

- Stoma counselling: Preoperative stoma counselling is essential to prepare the patient mentally and emotionally for life with a stoma. This support can be crucial in helping patients adapt to life with a stoma, ensuring they can manage their stoma effectively and maintain a good quality of life. People with planned stoma surgery and preoperative consultations with health care providers feel prepared for life with a stoma based on the information provided during their hospital stay [4]. Many IPAA patients will have previous experience with an ostomy, in case of previous subtotal colectomy or temporary diversion of the newly formed pouch. Discussing differences between end and loop ileostomy, as the potential for a more proximal stoma, is paramount, as understanding the patient experience with the previous stoma.
- Stoma site marking: Appropriate stoma site marking is crucial for preventing postoperative complications and ensuring optimal stoma function [5]. The marking process involves a thorough evaluation of the patient’s abdominal anatomy in multiple positions (sitting, standing, bending forward and lying supine). This assessment helps identify the most suitable location for the stoma, taking into account factors such as abdominal contours, skin folds, previous scars, hernias and the patient’s beltline. Following restorative proctocolectomy, patients may have midline scars, stoma closure site scars and even previous hernia repairs. Moreover, planning for more than one site is preferrable, due to the possibility of using a higher or contralateral site, to minimise tension on the small bowel mesentery. An appropriate site allows the patient to manage the stoma effectively, improving quality of life post-surgery. An experienced stoma care team is unvaluable for the patient and for the surgical team.
- Evaluation of surgical history: Prior surgical history is reviewed to understand the previous stages of pouch surgery, assess for any abdominal scars and consider whether the patient has had a stoma or hernia repair before. Note must be taken on any previous bowel resections, and presence of any anastomoses with its location and configuration (for example, ileostomy reversal). This is important for planning the surgical approach and siting the new stoma. History of previous intra-abdominal or pelvic sepsis and its management (drainage, endo-vacuum therapy) is defined, as it is the technique for construction of the original IPAA (stapled or hand-sewn) and management of the rectal stump at the time of emergency colectomy (in our practice, commonly left closed inside the peritoneal cavity, with trans-anal drainage facilitated by rectal examination and proctoscopy).
- Examination [6] under anaesthesia and pouchoscopy: A structured approach to ileoanal pouch assessment is required. Commenting is required on the presence of perianal disease, strictures at IPAA outlet and inlet, pouch configuration, pouch body, pre-pouch ileum and rectal cuff. Patients considered for defunctioning or pouch excision must undergo a pre-operative surgical assessment under anaesthetic, with intraoperative pouchoscopy and biopsies, in addition to imaging (MRI pelvis and small bowel).
- Assessment of residual small bowel: Preoperative imaging is used to estimate the length of the remaining small bowel, being aware of the risk for short bowel syndrome in these patients. Additionally, any existing pathology in the small bowel should be ruled out. The preferred technique in our department is magnetic resonance imaging, but CT enterography is also appropriate [7].

### Surgical technique

The surgical technique for diverting ileostomy involves careful attention to both the placement of the stoma and the preservation of bowel function for future procedures. The patient is placed in the modified Lloyd-Davies position to allow for access to the perineum for intraoperative pouchoscopy.

**Fig. 1:**
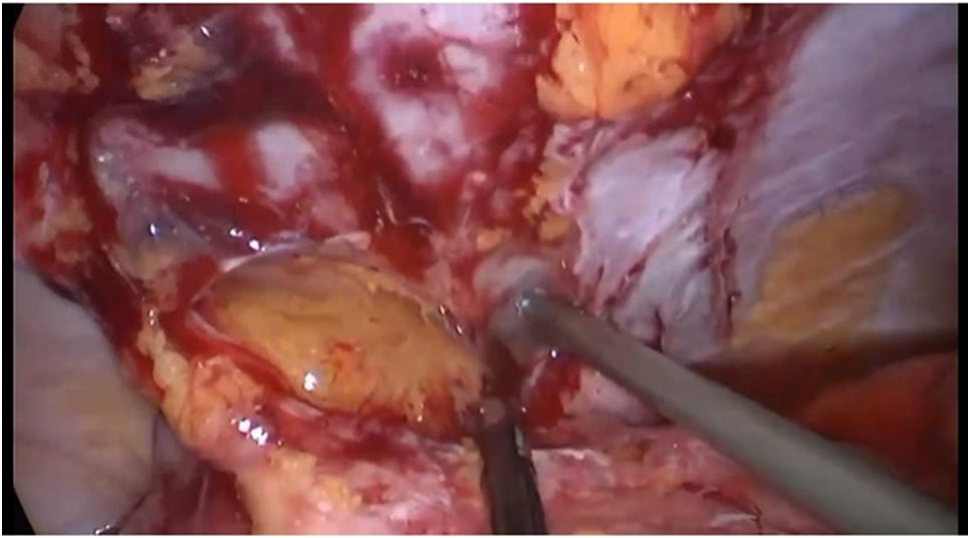
Hydro-dissection and cold sharp division of adhesions

Given the likelihood of small bowel adhesions from previous surgeries, laparoscopic access is recommended away from midline in patients with previous laparotomies and the Hasson technique is preferred over the Veress needle for pneumoperitoneum induction, often in the left iliac fossa. It is not our routine practice, but a direct approach with a full midline laparotomy is also acceptable. Adhesiolysis is performed using sharp dissection and hydro-dissection to minimize the risk of bowel injury (Fig. 1). A 5-mm camera facilitates use of all operating trocars to change position of the camera. A full small bowel walk-through confirms the length and quality of the remaining small bowel from duodeno-jejunal flexure to pouch inlet. Atraumatic graspers, avoiding manipulation of the small bowel mesentery, are preferred. Bowel length and location of any pathological findings is annotated, measured from the landmark of the duodeno-jejunal flexure.

**Fig. 2:**
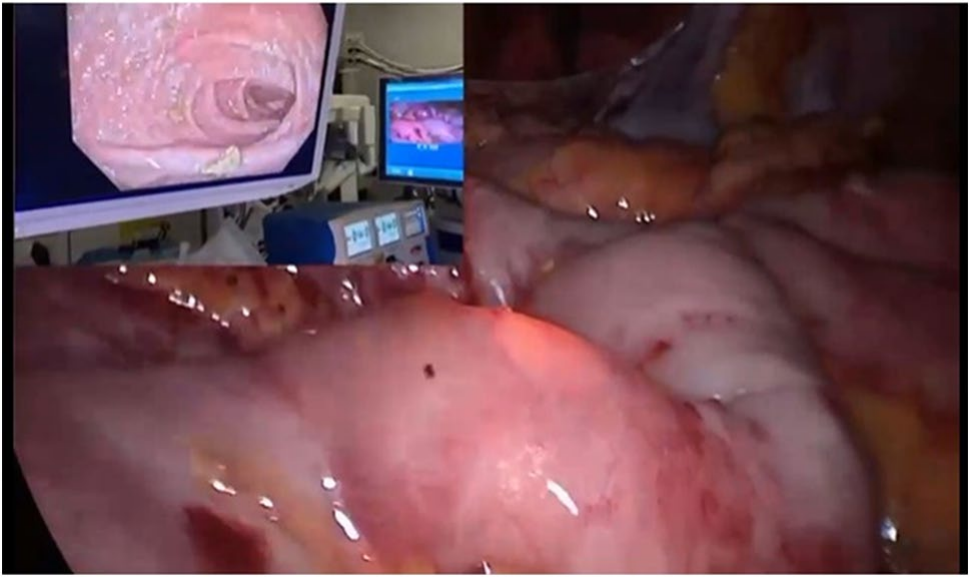
On table pouchoscopy to confirm pouch inlet and blind end locatio

Intraoperative pouchoscopy is conducted to assess the integrity of the pouch and to aid intra-operative localization of essential landmarks (Fig. 2). Structures such as the pouch body, inlet, pre-pouch ileum and blind-end are identified, and any previous ileostomy reversal anastomosis is noted, if present. Minimal insufflation of the pouch is required, suctioning the excess gas when completing to procedure, to avoid over distension of the bowel loops which would impair continuation of the laparoscopic approach.

**Fig. 3:**
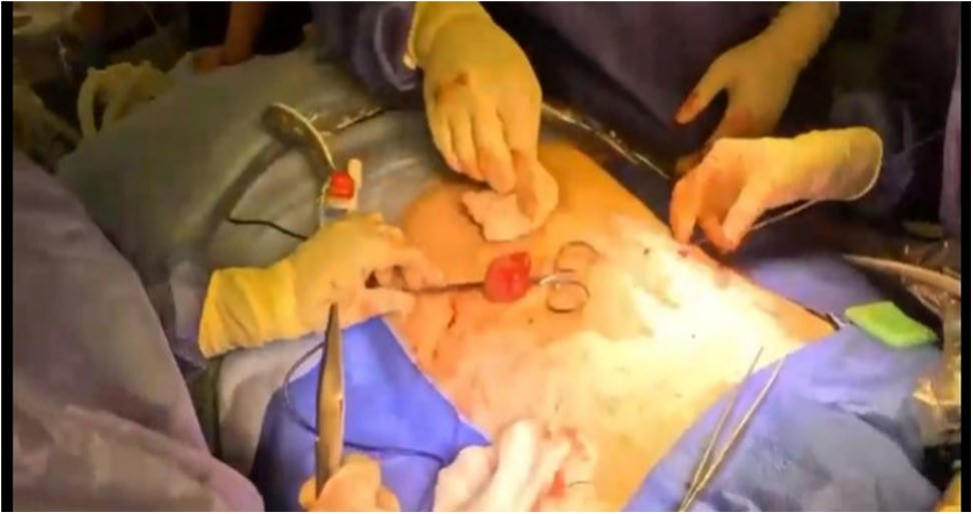
Tension-free loop ileostomy formation

The small bowel to be used for the ileostomy is approximated to the abdominal wall, ensuring it reaches without tension (Fig. 3). If the originally planned site does not allow this, an alternative pre-marked site is used. A loop ileostomy is preferred when reversal is possible (considering a loop-end if required to minimise tension), while an end ileostomy is preferred if a permanent stoma is planned. If a redo IPAA is planned, it is crucial that the ileostomy is no further than 20–40 cm from the pouch inlet, as a longer distance may compromise the possibility of a future redo IPAA. Similarly, for a permanent ileostomy, the most possible distal part of the ileum is used.

### Postoperative follow-up

Daily review of the stoma is carefully conducted by the surgical and stoma care team. Short-term postoperative care focuses on managing common surgical complications and high-output stoma, whilst long-term management is focused on persistent pouch dysfunction symptoms, such as anal discharge, pelvic pain or perianal disease. It is essential to prevent dehydration and renal failure. Initial treatment involves oral rehydration solutions and medications to reduce bowel motility and secretions [8]. Support from dietetics is advisable, to ensure appropriate intake and replacement of electrolytes. Ongoing monitoring post-discharge from hospital is required, with virtual and clinical reviews. In our unit, we collect patient-reported outcome measures, focused on psychological wellbeing, urogenital function, quality of life, fatigue and social functioning [9]. For patients with ongoing anal discharge due to poor pouch function, irrigation may be used to help empty the pouch more effectively. Pouch excision is required in patients who have severe on-going symptoms despite defunctioning and optimised medical therapy.

## Discussion

Ileostomy formation is a valid treatment for patients with severe pouch dysfunction, with or without associated excision of the ileoanal pouch. We describe a stepwise approach to preoperative management and intra-operative technique.

Ileal pouch excision is considered in cases where conditions such as severe sphincter insufficiency, pelvic or perianal sepsis and Crohn’s disease of the pouch cause significant symptoms. The procedure can be preceded by a diverting loop ileostomy, performed approximately 4 to 6 months prior to the definitive surgery [10]. Although technically more challenging, pouch excision, rather than pouch left in situ, is associated with a higher resolution of the symptoms leading to pouch dysfunction [11]. Nevertheless, the potential implications of long-term complications, such as persistent sinus cavity, urogenital and sexual dysfunction and infertility, need to be carefully balanced.

In a systematic review by Heuthorst et al., the rate of pouch failure (defined as the need for a permanent ileostomy with or without pouch excision) was found to be 7.8% after 5 years and 10.3% after 10 years [12]. Another recent meta-analysis of 26 studies involving 23,389 patients with ulcerative colitis who underwent restorative proctocolectomy showed an overall pouch failure rate of 6%, with an increase in failure from 5% in the first 10 years to 9% beyond 10 years of follow-up. These data are crucial for providing realistic expectations to patients, especially considering the significant physical and psychological impact of the disease [13]. Pouch excision has a high complication rate as reported by Kalaiselvan et al. with 31% postoperative complication rate and 11% of patients requiring reoperation [14].

Redo IPAA is a complex staged procedure, designed for selected patients who have experienced failure of their initial IPAA but still desire a functional ileal pouch. When feasible, salvaging the primary pouch is preferred to preserve bowel length and minimize the risk of short bowel syndrome [1, 15]. Salvage surgery, including redo IPAA, offers an alternative to pouch excision [16], despite salvage procedures being associated with increased morbidity and longer hospital stays compared to primary IPAA. The overall success rate of salvage procedures is estimated to be between 20 and 80%, but the rate of salvage is dependent on the duration of follow-up and the specific surgical technique used [17]. Theodoropoulos et al. conducted a systematic review of salvage procedures and found a 74% success rate, with 18% of patients eventually requiring pouch excision after redo IPAA [1]. While these numbers suggest that a significant proportion of patients can avoid permanent stomas, careful patient selection and meticulous surgical technique are essential for achieving favourable outcomes.

A continent ileostomy needs to be considered in patients having a pouch excision and a permanent stoma fashioned, as it can preserve the length of the ileal pouch by converting it in the continent ileostomy reservoir. A study by Lian et al. on continent ileostomy after failed ileal pouch-anal anastomosis reported a 30-day complication rate of 31.3% with no perioperative deaths. With a median follow-up of 5 years, the long-term dysfunction rate was 50%, the complication rate was 60.9%, and the revision rate was 45.3%, with a median quality-of-life score of 0.77, as assessed by the Cleveland Global Quality of Life Score [18]. Those results are confirmed by Ecker et al. In comparison to redo IPAA, continent ileostomy emerges as a valid option in selected cases, due to the high reoperation rate and surgical and stoma care expertise required [19].

The management of patients with a dysfunctional pouch is complex, and when a multidisciplinary recommendation for discussion of permanent defunctioning or excision is considered, careful surgical planning and technique is required. There is growing evidence to support the centralization of pouch surgery, particularly revisional procedures, in high-volume units. Concentrating these technically complex surgeries in specialized centres with dedicated perioperative care is essential for improving patient outcomes and allows teams to build the required expertise [20].

## Conclusion

Diverting ileostomy plays a critical role in managing severe ileoanal pouch dysfunction, particularly in cases where salvage surgery or permanent diversion is necessary. The choice of surgical approach should be guided by the underlying cause of pouch dysfunction, patient comorbidities and the potential for future restorative procedures.

## Supplementary Information

Below is the link to the electronic supplementary material.

Supplementary file1 (MP4 567799 KB)

## Data Availability

No datasets were generated or analysed during the current study.
